# Avocado Seeds: Extraction Optimization and Possible Use as Antioxidant in Food

**DOI:** 10.3390/antiox3020439

**Published:** 2014-06-10

**Authors:** Francisco Segovia Gómez, Sara Peiró Sánchez, Maria Gabriela Gallego Iradi, Nurul Aini Mohd Azman, María Pilar Almajano

**Affiliations:** 1 Chemical Engineering Department, Technical University of Catalonia, Avda. Diagonal 647, 08028 Barcelona, Spain; E-Mails: segoviafj@gmail.com (F.S.G.); sapeisa@yahoo.es (S.P.S.); maria.gabriela.gallego@upc.edu (M.G.G.I.); aini.azman@gmail.com (N.A.M.A.); 2 Chemical Engineering Department, Antonio José de Sucre National Experimental Polytechnic University, Avenida Corpahuaico, 3001 Barquisimeto, Venezuela

**Keywords:** RSM, avocado pit, ORAC, extraction, emulsion, oxidation, meat

## Abstract

Consumption of avocado (*Persea americana* Mill) has increased worldwide in recent years. Part of this food (skin and seed) is lost during processing. However, a high proportion of bioactive substances, such as polyphenols, remain in this residue. The primary objective of this study was to model the extraction of polyphenols from the avocado pits. In addition, a further objective was to use the extract obtained to evaluate the protective power against oxidation in food systems, as for instance oil in water emulsions and meat products. Moreover, the possible synergy between the extracts and egg albumin in the emulsions is discussed. In Response Surface Method (RSM), the variables used are: temperature, time and ethanol concentration. The results are the total polyphenols content (TPC) and the antiradical power measured by Oxygen Radical Antioxidant Capacity (ORAC). In emulsions, the primary oxidation, by Peroxide Value and in fat meat the secondary oxidation, by TBARS (Thiobarbituric acid reactive substances), were analyzed. The RSM model has an *R*^2^ of 94.69 for TPC and 96.7 for ORAC. In emulsions, the inhibition of the oxidation is about 30% for pure extracts and 60% for the combination of extracts with egg albumin. In the meat burger oxidation, the formation of TBARS is avoided by 90%.

## 1. Introduction

Vegetables and fruits are essential foods in our diet and also have many compounds that are beneficial for health due to minor components. These minor components include phenolic substances [1]. These are secondary metabolites of plants. They have an aromatic ring with one or more hydroxyl groups. Their complexity may be high, as for example quercetin, which is one flavone with several aromatic rings. The properties depend on the arrangement and/or structure of the molecule [2].

In recent times, many plants have been studied in order to characterize them depending on the amount of polyphenols they have and on their potential use [3].

The polyphenols are associated with the potential prevention of diseases which are due to the presence of free radicals, such as cardiovascular insufficiency, hypertension, inflammatory conditions, asthma, diabetes and Alzheimer’s [4], thanks to their antiradical power. For this reason, they are very useful in food products, since they prevent lipid peroxidation due to the attack of free radicals [5]. They also protect against oxidation, direct or indirect, caused by metal cations [6]. These cations stimulate the creation of reactive oxygen species (ROS), which are harmful to the health. In some cases, polyphenols have been used as preservatives, protecting against microorganisms [7].

The process of food, especially for IV and V gamma products, produces many byproducts and waste. This type of waste has a significant environmental impact due to the organic charge. It also has associated handling, transport and storage costs, among others. Therefore, more and more alternative uses for these residues are sought, as for instance animal feed and fertilizers, among others. In the present case, it is interesting to obtain, through an optimized extraction process, harmless substances with high antioxidant power. Thus, what was a waste becomes a “high value-added” product [8,9]. Previous examples already studied [10,11,12] are the orange juice industry, where a large amount of skin and seeds are produced with a high content of polyphenols and the industry of processed apple, pear and peach, with a significant amount of skin byproduct. There is evidence that the skin may even have a greater amount of polyphenols than the flesh [13]. Also, the waste from wine and beer production includes phenolic compounds [9]. Other studies have focused on the shells of nuts, rice and wheat in which large amounts of polyphenols are found [14].

In the avocado industry the pulp is used, while the skin and the seeds are discarded as waste. These residues are rich in polyphenols with antioxidant and antimicrobial power [15]. Among the polyphenols the (+)-catechin and (−)-epicatechin [16] and chlorogenic and protocatechuic acid, are included [14]. Previous studies on this residue have been applied to pork burgers and have been shown to be effective in preventing oxidation and microbial growth [15].

Given the above, it can be concluded that polyphenols obtained from these industrial wastes can be potent antioxidants and, in some cases, they are better than synthetic antioxidants such as BHA or BHT which in high doses can become toxic [17].

In order to optimize the extraction process, response surface methodology (RSM) has been used. Phenolic compounds extraction optimization from strawberries [18], apple pulp [9] and residues of chestnuts [19], are examples of this. This method establishes a multivariable mathematic model to obtain the relationship between responses and independent variables [20,21] with the use of a minimal number of experiments.

This paper consists of two main objectives. First, a mathematical model was obtained to predict the best conditions of extraction of polyphenols from dried avocado seed. Second, an extract using these conditions was obtained and the effect of lyophilized powder in the delay oxidation in oil-in-water (O/W) emulsions and beef meat burgers analyzed.

## 2. Experimental Section

### 2.1. Materials

2,2′-Azo-bis(2-amidinopropane) dihydrochloride (AAPH), was used as peroxyl radical source. Trolox (6-hydroxy-2,5,8-tetramethylchroman-2-carboxylic acid), ethanol, fluorescein, AAPH, BHA, egg albumin, *p*-anisidine (4-amino-anisole; 4-methoxy-aniline), isooctane, potassium persulfate, acetic acid (glacial) and 2-thiobarbituric acid were purchased from Sigma-Aldrich Company Ltd. (Gillingham, UK). Folin-Ciocalteu reagent, sodium carbonate and 1,6-diaminohexane were supplied by Merck (Darmstadt, Germany). Trichloroacetic acid, hydrochloric acid and Tween^®^ 20 (Panreac Química S.L.U, Barcelona, Spain) were acquired from Panreac Química S.L.U. (Barcelona, Spain). Refined sunflower oil, with no added antioxidants, was purchased from a local retail outlet. All compounds were of reagent grade.

### 2.2. Avocado Preparation

The avocado (*Persea americana*) was obtained in the local market; the seeds were separated from other edible parts. This waste was homogenized and frozen at −80 °C for lyophilization. Then the seeds were ground into a powder by using a Moulinex mill (A5052HF, Moulinex, Lyon, France). The particle size was standardized with a number 40 mesh sieve. Finally, the powder was stored in a dark bottle in a desiccator until use.

### 2.3. Extraction Procedure

Extraction was carried out in dark bottles: lyophilized sample powder (0.25 g) was blended with 15 mL of solvent of concentration specified by the experimental design (Table 1). This mixture was placed in a bath by stirring at the required temperature and time specified by the experimental design (Table 1). At the end, it was cooled in a refrigerator at 5 °C, centrifuged (Orto Alresa, Madrid, Spain) at 2500 rpm for 10 min, vacuum filtered and the lost solvent was replaced. The extract was stored at −20 °C until used for analysis.

### 2.4. Total Phenolic Content (TPC)

TPC was determined spectrophotometrically following the Folin-Ciocalteu colorimetric method [22]. A sample diluted 1:4 with milli-Q water was stirred in triplicate. The final concentration in the well (96 wells plate was used) was: 7.7% v/v sample, 4% v/v Folin-Ciocalteu’s reagent, 4% saturated sodium carbonate solution and 84.3% of milli-Q water, all mixed. The solution was allowed to react for 1 h in the dark and the absorbance was measured at 765 nm using a Fluostar Omega (BMG Labtech, Ortenberg, Germany). The total phenolic content was expressed as mg Gallic Acid Equivalents (GAE)/g dry weight.

**Table 1:** Experimental design and responses for extraction.

Temperature (°C)	Ethanol Concentration (%)	Time (min)	TPC (mg GAE/g dw)	ORAC (mg TE/g dw)
60.00	60.00	25.00	41.00 ± 0.97	104.16 ± 2.13
60.00	93.63	25.00	35.10 ± 0.24	116.12 ± 1.03
80.00	80.00	5.00	46.78 ± 0.59	153.17 ± 3.84
26.36	60.00	25.00	40.78 ± 0.17	70.54 ± 0.97
60.00	60.00	25.00	41.10 ± 0.57	106.10 ± 2.40
40.00	40.00	45.00	43.24 ± 0.76	104.01 ± 2.35
80.00	80.00	45.00	45.43 ± 0.49	144.94 ± 2.84
80.00	40.00	45.00	45.37 ± 1.39	130.08 ± 2.65
80.00	40.00	5.00	43.70 ± 0.66	150.03 ± 1.73
60.00	60.00	25.00	40.90 ± 0.47	104.28 ± 1.03
60.00	60.00	55.22	42.87 ± 0.70	158.77 ± 1.33
40.00	40.00	5.00	41.19 ± 0.55	99.17 ± 1.81
60.00	60.00	2.77	42.92 ± 1.13	155.44 ± 2.71
93.64	60.00	25.00	46.95 ± 0.09	126.23 ± 3.35
60.00	26.36	25.00	42.33 ± 0.10	129.78 ± 3.84
40.00	80.00	45.00	38.98 ± 0.45	100.72 ± 3.27
40.00	80.00	5.00	35.48 ± 0.55	91.01 ± 3.51

GAE: Galic Acid Equivalents; TE: Trolox Equivalents; TPC: Total Phenolic Content; ORAC: Oxygen Radical Antioxidant Capacity.

### 2.5. ORAC Assay

Antioxidant activities of avocado seeds extracts were determined by the ORAC assay, as reported by Casettari *et al.* [23]. The assay was carried out using a Fluostar Omega equipped with a temperature-controlled incubation chamber. The incubator temperature was set to 37 °C. The extract samples were diluted 1:20 with milli-Q water. The assay was performed as follows: 20% of sample was mixed with Fluorescein 0.01 mM, and an initial reading was taken with excitation wavelength, 485 nm and emission wavelength, 520 nm. Then, AAPH (0.3 M) was added, measurements were continued for 2 h every 5 min. This method includes the time and decrease of fluorescence. The area under the curve (AUC) was calculated. A calibration curve was made each time with the standard Trolox (500, 400, 250, 200, 100, 50 mM). The blank was 0.01 M phosphate buffered saline (pH 7.4). ORAC values were expressed as mg Trolox Equivalents (TE)/g of dry weight.

### 2.6. Statistical Analysis

RSM was used to determine the optimal conditions of polyphenol extraction. A central composite design (CCD) was used to investigate the effects of three independent variables with two levels (solvent concentration, extraction temperature, and extraction time) with the dependent variables (TPC, ORAC activity). CCD uses the method of least-squares regression to fit the data to a quadratic model.

The adequacy of the model was determined by evaluating the lack of fit, coefficient of determination (*R*^2^) obtained from the analysis of variance (ANOVA) that was generated by the software. Statistical significance of the model and model variables were determined at the 5% probability level (α = 0.05). The software uses the quadratic model equation shown above to build response surfaces. Three-dimensional response surface plots and contour plots were generated by keeping one response variable at its optimal level and plotting that against two factors (independent variables). Response surface plots were determined for each response variable. The coded values of the experimental factors and factor levels used in the response surface analysis are shown in Table 1. The graphics and the RSM analysis were made by software Matlab version R2013b (The MathWorks Inc., Natick, MA, USA, 2013). All responses were determined in triplicate and are expressed as average ± standard deviation. The answers have a percentage deviation less than 10%.

### 2.7. Water-Oil Emulsions

Oil-in-water emulsions (20.2 g) were prepared by dissolving Tween-20 (1%) in acetate buffer (0.1 M, pH 5.4), either with or without protein, namely egg albumin (0.2% w/w) and avocado seeds extracts (0.45% w/w, 0.225% w/w, 0.1125% w/w). The emulsion was prepared by the dropwise addition of oil (sunflower oil) to the water phase, cooling it in an ice bath with continuous sonication with a Vibracell sonicator (Sonics and Materials, Newtown, CT, USA) for 4 min. All emulsions were stored in triplicate in 60 mL glass beakers in the dark (inside an oven) at 30 °C in an incubator. Two aliquots of each emulsion (0.005–0.1 g, depending on the extent of oxidation) were removed periodically for determination of peroxide value (PV).

### 2.8. Peroxide Value (PV)

PV was determined by the ferric thiocyanate method [24] (after calibrating the procedure with a series of oxidized oil samples analyzed using the AOCS Official Method Cd 8-53). Data from the PV measurements were plotted against time.

### 2.9. Meat Preparation

Fresh beef meat was purchased from a local processor 96 h postmortem. All subcutaneous and inter-muscular fat and visible connective tissue were removed from the fresh beef muscle. Lean meat was ground through Ø-4 mm plate using a meat grinder (PM-70, Mainca, Barcelona, Spain). The ground meat was divided into six portions for each experiment prior to the addition of the sodium chloride or different concentration of powder (freeze-dried extract of powder of avocado). The lyophilized avocado and the powder of direct avocado were mixed with the salt final concentration of 1.5% (w/w). Each portion of beef meat was mixed manually with each solid. Each mixed sample was divided into nine smaller portions (about 10 g each) and allocated onto trays. The meat was packed under MAP (20% CO_2_ and 80% O_2_) in polystyrene/EVOH/polyethylene trays, heat sealed with laminated barrier film and stored at 4 ± 1 °C for 8 days. Patties were evaluated for lipid oxidation.

### 2.10. Thiobarbituric Reactive Substances

Fat meat oxidation was determined by the concentration of thiobarbituric acid-reactive substances (TBARS) using the method described by Domenech Asensi (2013) [25] with some modifications. In the dark, 1 g of burger patty was dispensed in tubes and 1 mL of EDTA was added. The samples were homogenized for 5 min sin an Ultra-Turrax (Ika^®^-Werke, Staufen, Germany) with 5 mL of TBARS reactive (Trichloroacetic acid, 9.2%; Hydrochloric acid, 2%; Thiobarbituric acid, 0.22%, all w/w final). During homogenization, the tubes were placed in an ice bath to minimize the development of oxidative reactions. The sample tubes were heated at 90 °C in a boiling water bath for 20 min and then left to cool. Two milliliters of slurry was centrifuged (10,000 rpm for 10 min). The absorbance was measured at 531 nm in a Spectrophotometer Zuzi model 4201/20 (AUXILAB, SL, Navarra, Spain). The result is expressed in mg of MDA/kg sample.

## 3. Results and Discussion

### 3.1. Extraction Optimization

Experimental design was carried out to see the effects of temperature, solvent concentration (ethanol) and time in both TPC and radical scavenging (measured by ORAC). Several authors used ethanol/water as solvent to extract different raw material polyphenols, such as seeds, grape marc, fruits, among others [26,27,28,29,30]. Ethanol concentration with the highest polyphenols yield is in the range of 10%–60%. Ethanol, instead of methanol, is used when it is necessary to reduce the toxicity of extracts [18]. The time effect was measured between 5 and 45 min, because some research reported that it is enough to achieve the maximum amount of polyphenols [31,32]. Temperature bounds were taken between 40 and 80 °C, to achieve the maximum temperature that does not have a negative effect on the polyphenols stability [33]. All these parameters are collected in Table 1 which shows the experimental design for the variables temperature (T), ethanol concentration (% EtOH) and time (*t*), with responses of TPC and antiradical activity measured by ORAC.

**Figure 1:**
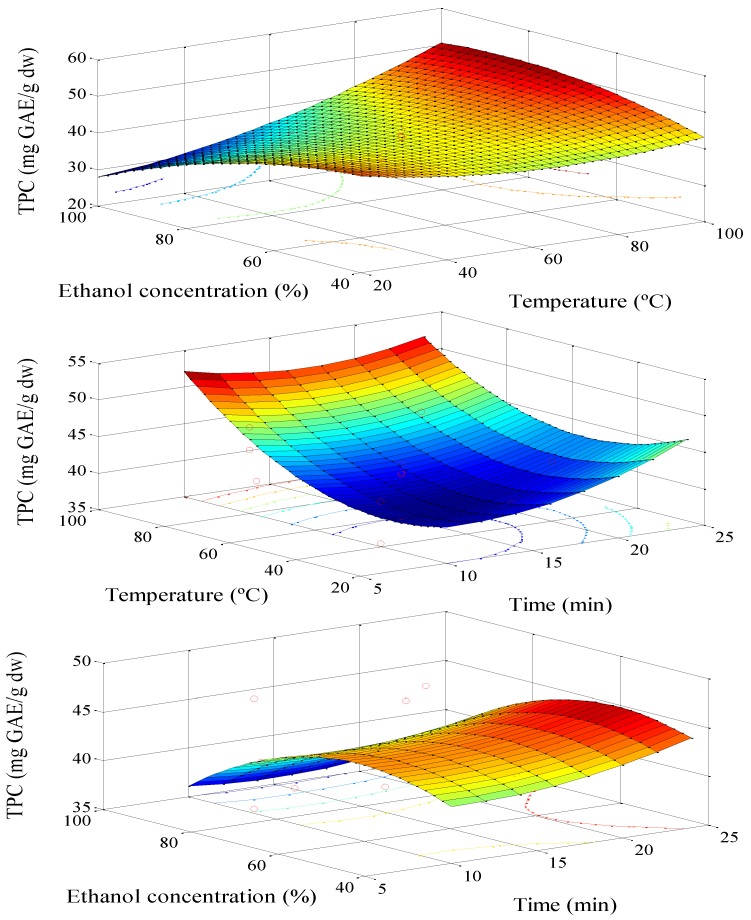
Response surface model plot: the variable is the total phenolic content (TPC) of the extract. % EtOH with temperature; temperature with time; % EtOH with time.

Figure 1 shows the relationship between the variables *T*, % EtOH and t in polyphenol extraction. The process is favored by high temperatures and low concentrations of ethanol (in the studied range). This behavior can be attributed to the nature of the polyphenols present in the sample, mainly chlorogenic acid and protocatechuic acid [34] both highly soluble in water. The solvent plays an important role in mass transfer of the compounds; not all polyphenols show identical behavior in the extraction process, and the less polar polyphenols are favored by the highest concentration of ethanol [9].

The effect of temperature on the extraction is associated with the solubility of the components present in the avocado pit. This variable, *T*, has a marked influence on the diffusivity of the substances [30]. Solubility increases with temperature. Time has no influence in the extraction process. This means that from the beginning, the extraction is governed by the solubility and diffusion, and both are almost complete after 5 min.

**Figure 2:**
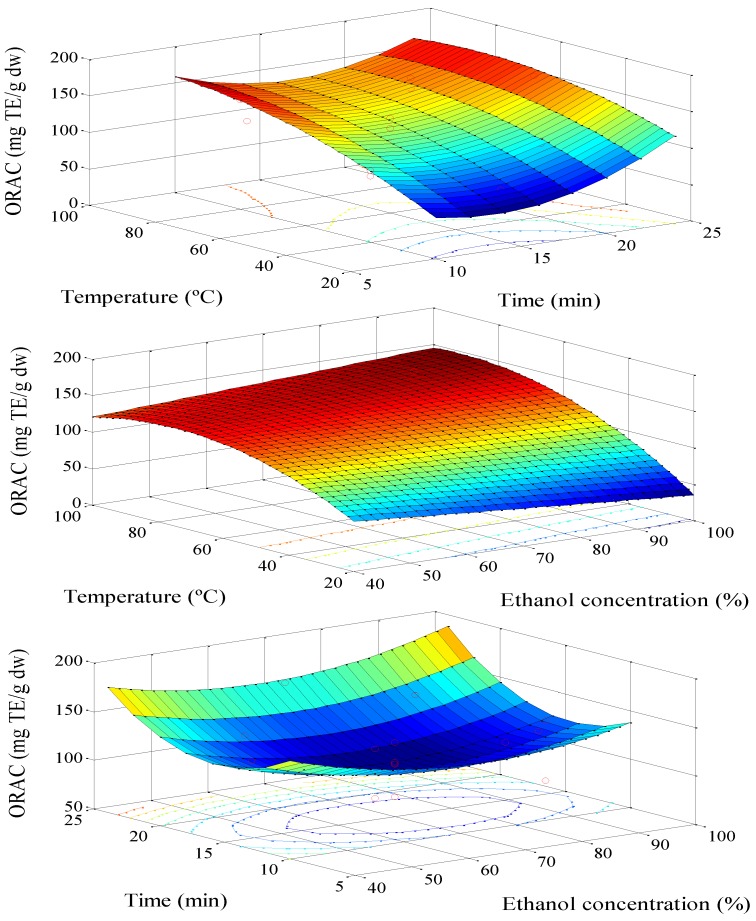
Response surface model plot: the variable is the Oxygen Radical Antioxidant Capacity (ORAC) of the extract. Temperature with time; % EtOH with temperature; % EtOH with time.

Figure 2 shows the effect of the parameters on the antioxidant power measured by ORAC. The ORAC increases with temperature. In the investigated range, the ORAC is increased about 44% (Table 1). Furthermore, as stated above, it is in accordance with the higher polyphenols solubility at high temperature. This means that these kinds of polyphenols are thermo-resistant [20].

The effect observed for the percentage of ethanol is similar to that described in the TPC. An increase in the ethanol concentration causes a decrease in antioxidant activity. It is not a new fact, because similar results were described in other studies and were justified by the polarity of the compounds of the extract [18].

On TPC the variable t has no influence, while on ORAC small changes were observed, but all of them with similar final values. One possible explanation is that there are antioxidant compounds with slow solubilization and, therefore, the time promotes an increase in total extraction [35].

Table 2 shows the “*p* values” of the mathematical model for the coefficients, with the decoded variables. It starts with the complete model, taking the variables that have less influence, *i.e.*, with *p* > 0.05. For TPC all those that are with % EtOH and t are involved. This means that the more important variable is T. However, on the ORAC, the variables that have more influence are % EtOH, *t*, and these quadratic terms. From the data, different iterations were made and less influential terms were eliminated; after which the values were recalculated. With these data the reduced model was obtained and provided a better fit. In ORAC the predicted *R*^2^ becomes 77.88 which is within the range of a good set [36].

Therefore, with the exception of *T* × % EtOH for the TPC, all of the crossed terms disappear in the reduced model (which is used to adjust and to determine the optimal extraction conditions).

Additionally, the quadratic variables % EtOH × % EtOH and *t* × *t*, as well as the linear variable *t* are eliminated for the TPC. The quadratic term *T* × *T* is eliminated from the model which determines the scavenging activity. This is summarized in Table 2.

Table 3 lists the completed model and the reduced model equations. The reduced model has a higher *R*^2^ predicted which means that it is more reliable in estimating a response.

When the fitting was considered good enough, the experiment was performed in the laboratory to obtain the real value. Table 4 contains these values for the TPC and for the ORAC. The TPC is fitted with less than a 4% error (the predicted value is 43.6 mg GAE/g dw, compared to an experimental value of 45.01 mg GAE/g dw). This indicates that the initial hypothesis was correct, and demonstrates that T is the variable with the greatest influence on the maximum TPC extraction.

**Table 2:** *p*-Values for each of the constants in the equation of the mathematical model.

Term	*p*-Value	
	Response	
	TPC	ORAC
**Complete Model**		
Constant	0.001	0.006
Temperature (°C)	0.012	0.069
Ethanol (%)	0.291	0.022
Time (min)	0.804	0.001
Temperature (°C) × Temperature (°C)	0.014	0.135
Ethanol (%) × Ethanol (%)	0.622	0.046
Time (min) × Time (min)	0.068	0.000
Temperature (°C) × Ethanol (%)	0.003	0.186
Temperature (°C) × Time (min)	0.119	0.071
Ethanol (%) × Time (min)	0.610	0.435
**Reduced Model**		
Constant	0.000	0.000
Temperature (°C)	0.005	0.000
Ethanol (%)	0.001	0.031
Time (min)	-	0.000
Temperature (°C) × Temperature (°C)	0.029	-
Ethanol (%) × Ethanol (%)	-	0.033
Time (min) × Time (min)	-	0.000
Temperature (°C) × Ethanol (%)	0.004	-

TPC (mg GAE/g dw); ORAC (mg TE/g dw); GAE: Gallic Acid Equivalent; TE: trolox equivalent.

However, the values which maximize scavenging activity (ORAC) have a greater deviation. The value predicted by the reduced model was 200.66 mg TE/g dw, compared to an experimental value of 154.3 mg TE/g dw, which represents a deviation of 23.1%.

The best-fitting experimental conditions were then applied, *i.e.*, 23 min extraction with 56% EtOH and 63 °C. This extract was lyophilized and used in subsequent experiments.

**Table 3:** Mathematical equations from Response Surface Method (RSM) for each of the responses, with their respective value of *R*^2^ and *R*^2^-predicted.

Response	Equation	*R*^2^ Value	
		*R*^2^	*R*^2^ Pred.
**Complete Model**			
TPC	62.87 − 0.47 *T* − 0.25 [%] − 0.14 *t* + 0.003 *T*^2^ − 0.001 [%]^2^ + 0.03 *t*^2^ + 0.006 *T* × [%] − 0.007 *T* × *t* − 0.003 [%] × *t*	94.69	57.0
ORAC	318.2 + 2.03 *T* − 04.41 [%] − 019.5 *t* − 00.009 *T*^2^ + 0.023 [%]^2^+ 0.7 *t*^2^ + 0.012 *T* × [%] − 00.053 *T* × *t* − 00.03 [%] × *t*	96.7	75.0
**Reduced Model**			
TPC	69.7 − 00.53 *T* − 00.39 [%] + 0.002 *T*^2^ − 00.006 *T* × [%]	85.7	66.76
ORAC	345.7 + 1.01 *T* − 03.92 [%] − 022.01 *t* + 0.027 [%]^2^ + 0.73 *t*^2^	91.88	77.88

*T*: Temperature (°C); [%]: Ethanol concentration (%); *t*: Time (min); Pred.: response predicted by model. TPC in mg GAE/g dw and ORAC in mg TE/g dw.

**Table 4:** Optimal conditions for the extractions for TPC and ORAC, given by RSM.

Model	Conditions			Response		
	Temperature (°C)	Ethanol (%)	Time (min)	Predicted	Predicted RM	Experimental
**TPC**	63	56	23	51.75	43.6	45.01
**ORAC**	93.6	44.7	7	206.82	200.66	154.3

TPC in mg GAE/g dw; ORAC in mg TE/g dw.

### 3.2. Extract Optimized Effect in Oil-in-Water Emulsions (O/W)

**Figure 3:**
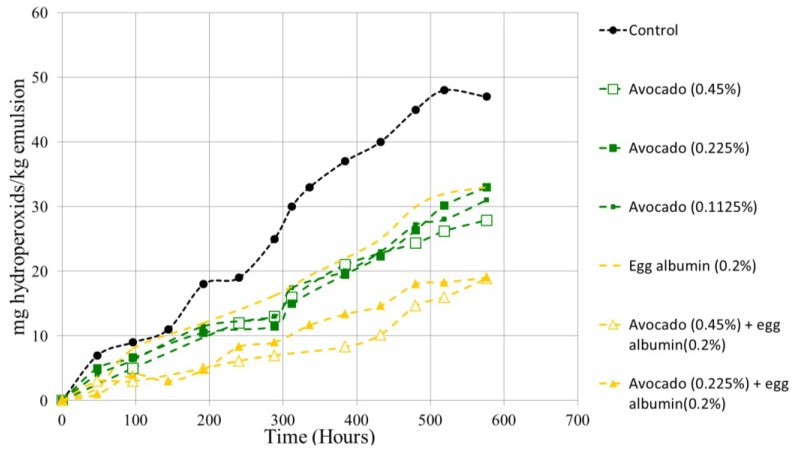
Peroxide values *vs.* time in the emulsions.

Figure 3 shows the evolution of peroxide value over time. In this case, the possible synergy between the extract (with different concentrations) and egg albumin was determined. Firstly, it should be noted that both albumin and various concentrations of the extract of avocado produce significant protection against oxidation. For example, within the 400 h of the experiment the amount of hydroperoxides produced is 90% higher in the control than in any of the samples (20 mg hydroperoxides/kg of emulsion hydroperoxides *vs.* 38 mg/kg for the emulsion control). Notably, there were no significant differences (*p* < 0.05) for the three tested avocado concentrations (0.1125%, 0.225% and 0.45% w/w), as well as egg albumin (0.2% w/w). This fact could be explained by the solubility of the lyophilized extract in water and the ability to coat the oil drop generated in the emulsion and prevent oxidation thereof. The necessary concentration that allows this protection is already achieved with 0.1125% and the results do not improve if increased. Similar behavior has been published elsewhere [37,38,39].

In fact, putting together two different compounds (avocado pit extract and egg protein) allows greater protection against oxidation and further differentiates the two concentrations of the tested extract. For example, the time required to reach 15 mg hydroperoxides/kg emulsion goes from 180 h of the control group up to 480 h for the sample containing 0.45% extract + 0.2 egg protein. This is an increase of 260% superior durability. In the intermediate areas three avocado extract concentrations were tested, as well as the protein (an increase in the durability between 150% and 180%) and one that contains 0.225% of avocado and 0.2% protein, with an improvement of the durability of 220%. Almajano and Bonilo-Carbognin already published similar results of synergy [40,41]. As a summary, it can be said that increasing the concentration of the extract does not improve the durability. However, the incorporation of small amounts of protein allows significant differences to be found between the samples containing protein and those that do not contain it.

Avocado pits contain polyphenolic compounds (such as protocatechuic acid, chlorogenic acid, syringic acid and rutin), which are very strong antioxidants [34]. In 2010, Sasaki [42] studied the antioxidant power of chlorogenic acid in oil-water emulsions. The effects discovered are remarkable. The authors analyzed the presence of other compounds, which in that case were also polyphenolic compounds. Additionally, they demonstrated that the presence of several different compounds provided better results than the added individual effects.

As it was stated before, 1% of surfactant (Tween-20) was added to the emulsion prepared in the present work. This eases the dissolution of the polyphenolic compounds, thus increasing the antioxidant activity in the emulsion.

### 3.3. Effect of the Extract in Burger Meat

**Figure 4:**
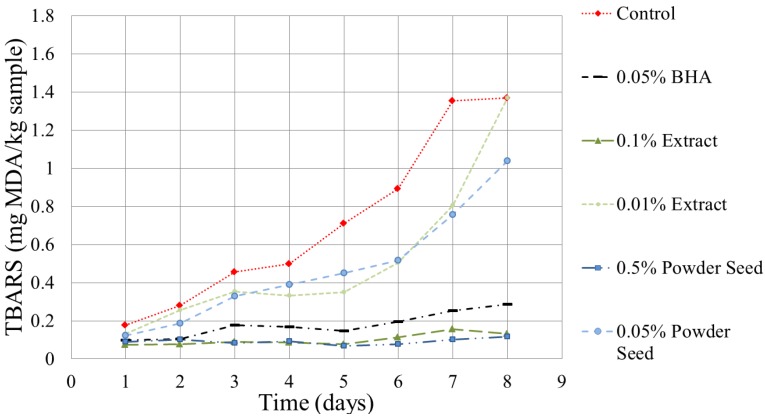
The TBARS (Thiobarbituric acid reactive substances) values for the meat emulsions.

The TBARS method is widely used to determine the oxidation of fats and oils in foods [43,44,45]. In Figure 4, the evolution of TBARs *vs.* time for each of the studied beef burger meat patties is collected. Samples containing 0.1% lyophilized extract and 0.5% direct seed powder have no significant differences compared with the BHA (0.05%), but show a big difference compared with the control. The lower concentration (0.01% and 0.05% lyophilized extract powder direct seed) presented intermediate behavior, as expected. The duration of the experiment was 8 days and it was observed that the burger meat with 0.5% seed powder and 0.1% of lyophilized extract had no significant oxidation, or the protection is higher than 90%. These results are similar to those reported by Weiss *et al.* [46] for pork burgers. That study examined protecting fat oxidation also with excellent results [46]. Additional results along the same lines have avocado oil added directly to the pork burgers. This shows a positive effect on the conservation of the burger [47].

It is not the first time avocado pits have been used in meat products. Rodríguez-Carpena *et al.* (2011) [15], prepared pork meat pies and inserted the grinded avocado pits to protect the meat against lipid oxidation. The authors indicated that one of the factors might be the formation of chelates with the copper and iron cations. These cations, in their free ionic state, could cause the creation of free radicals.

## 4. Conclusions

RSM was used to identify the best conditions for the extraction of compounds with an antioxidant activity from an organic residue: the avocado pit. The reduced model obtained provides parameters that fit with those of the TPC (with a 3.13% error when compared to the experimental value).

The lyophilized extract was used as protection from the oxidation of oils (oil-in-water emulsions) and fat (beef burgers) with excellent results, especially in meat, in which the durability of the burger meat is significantly increased relative to oxidation.

These studies should encourage further exploration in this area of study in order to obtain a byproduct of the natural antioxidants that currently as waste are worthless.
